# Identification of a New Target of miR-16, Vacuolar Protein Sorting 4a

**DOI:** 10.1371/journal.pone.0101509

**Published:** 2014-07-17

**Authors:** Neeta Adhikari, Weihua Guan, Brian Capaldo, Aaron J. Mackey, Marjorie Carlson, Sundaram Ramakrishnan, Dinesha Walek, Manu Gupta, Adam Mitchell, Peter Eckman, Ranjit John, Euan Ashley, Paul J. Barton, Jennifer L. Hall

**Affiliations:** 1 Lillehei Heart Institute, Department of Medicine, University of Minnesota, Minneapolis, Minnesota, United States of America; 2 Division of Biostatistics, University of Minnesota, Minneapolis, Minnesota, United States of America; 3 Department of Surgery, Cardiothoracic Surgery Division, University of Minnesota, Minneapolis, Minnesota, United States of America; 4 University of Minnesota Genomics Center, University of Minnesota, Minneapolis, Minnesota, United States of America; 5 Department of Pharmacology, University of Minnesota, Minneapolis, Minnesota, United States of America; 6 Center for Public Health Genomics, University of Virginia, Charlottesville, Virginia, United States of America; 7 National Institute of Heart Research, Cardiovascular Biomedical Research Unit, Royal Brompton and Harefield Trust, London, United Kingdom; 8 Center for Inherited Cardiovascular Disease, Department of Medicine, Stanford University, Stanford, California, United States of America; 9 Heart Science Centre, National Heart and Lung Institute, Imperial College, London, United Kingdom; Texas A & M, Division of Cardiology, United States of America

## Abstract

**Rationale:**

The rationale was to utilize a bioinformatics approach to identify miRNA binding sites in genes with single nucleotide mutations (SNPs) to discover pathways in heart failure (HF).

**Objective:**

The objective was to focus on the genes containing miRNA binding sites with miRNAs that were significantly altered in end-stage HF and in response to a left ventricular assist device (LVAD).

**Methods and Results:**

BEDTools v2.14.3 was used to discriminate SNPs within predicted 3′UTR miRNA binding sites. A member of the miR-15/107 family, miR-16, was decreased in the circulation of end-stage HF patients and increased in response to a LVAD (p<0.001). MiR-16 decreased Vacuolar Protein Sorting 4a (VPS4a) expression in HEK 293T cells (p<0.01). The SNP rs16958754 was identified in the miR-15/107 family binding site of VPS4a which abolished direct binding of miR-16 to the 3′UTR of VPS4a (p<0.05). VPS4a was increased in the circulation of end-stage HF patients (p<0.001), and led to a decrease in the number of HEK 293T cells *in vitro* (p<0.001).

**Conclusions:**

We provide evidence that miR-16 decreases in the circulation of end-stage HF patients and increases with a LVAD. Modeling studies suggest that miR-16 binds to and decreases expression of VPS4a. Overexpression of VPS4a decreases cell number. Together, these experiments suggest that miR-16 and VPS4a expression are altered in end-stage HF and in response to unloading with a LVAD. This signaling pathway may lead to reduced circulating cell number in HF.

## Introduction

MiRNAs regulate gene function in heart development, physiology and pathology. The miRNA–mRNA interaction is affected by the presence of single nucleotide polymorphisms (SNPs). A SNP within or near the miRNA binding sites of targets has been shown to influence the susceptibility to disease [1], [2]. In this study we identified new miRNA/RNA targets using a novel approach of utilizing conserved SNPs in miRNA binding regions as a roadmap. Determining how short RNAs (miRNA and siRNA) impact mRNA target expression within single cells and how this process influences disease initiation and progression is still not known. Recent work has suggested that within each cell, 85% of genes contain fewer than 100 copies [3]. Mullokandov et al provide evidence that only the most abundant miRNA regulate target gene expression [4]. The turnover rate of miRNA has been shown to alter gene expression [5], as well as the secondary structure of the target gene [6].

Several publications have cited circulating expression of miRNA in end-stage HF; however it is challenging to find how the levels of these miRNA compare to others in the circulation [7]–[11]. These studies identified increases in miR-423-5p and miR-499, and a decrease in miR-126 [7]–[11]. MiR-16 is one of the highest expressed mRNAs in the circulation. Expression of miR-16 is widely known to be high in all circulating cells [12]. Reticulocytes (immature red blood cells) and platelets exhibit the highest expression of miR-16 [12]. The role of miR-15/107 family members, including miR-16 in peripheral blood in the context of HF has not been defined. MiR-15 -16, -195, -497, -103, -107, -503, share a common sequence “AGCAGC” starting at the first or second nucleotide from the 5′ mature end of the miRNA [13]. This evolutionary conserved family of miRNAs targets a disproportionately high number of cell cycle regulatory genes [14]–[18]. A predicted target, Vacuolar Protein Sorting 4a, VPS4a, was identified with a SNP rs16958754 in the miR-15/107 family binding site. VPS4a is an ATPase and is one of many vacuolar protein sorting associated proteins involved in diverse cellular processes including multivesicular body formation, cytokinesis, and endosomal trafficking [19].

In this study, we identify miR-16 as the specific member of the miR-15/107 family that decreases in peripheral blood of patients with end stage HF and increases significantly one week post ventricular assist device (LVAD) therapy. The novel target of miR-16, VPS4a that we identified and validated *in vitro*, increases in peripheral blood of end stage HF patients and decreases one week following LVAD therapy. MiR-16 has been identified in CD45 and CD14 positive cells [12]. Overexpression of VPS4a significantly inhibited total cell number, suggesting that the miR-16-VPS4a pathway inhibits proliferation of CD45 and CD14 positive cells in end stage HF.

## Methods

An expanded methods section (Text S1) is available as Supporting Information.

### Ethics statements

All procedures followed were in accordance with the ethical standards of the responsible committees on human experimentation. Written consents were obtained from eligible patients for being included in this study. Samples were collected from consenting participants and the study was conducted under the Institutional Review Board approval from the University of Minnesota, USA the Royal Brompton and Harefield Cardiovascular Biomedical Research Unit, UK and Stanford University. All samples used in the study and the original repository are listed in Table S5.

### Heart samples

Heart samples from patients with end-stage HF were collected at the time of LVAD implant (HF, n = 10) at the University of Minnesota. Non-failing control heart samples (Control, n = 9, 45±5, 57% male) were obtained from Dr. Euan Ashley, Stanford University.

### Peripheral blood samples

Blood samples from patients with end-stage HF (n = 9) were collected prior to LVAD implant and 7 days post-implant (n = 9) as described [20] (Table 1). Blood samples from healthy gender and age matched subjects were labeled as controls (n = 6, average age 54±4, 60% male, 83% Caucasian).

**Table 1:** Characteristics of patients with end-stage HF. Blood samples were obtained prior to LVAD implantation (n = 9) and 7 days post implant (n = 9).

Patient ID	1	2	3	4	5	6	7	8	9
**Age**	62	65	54	46	63	76	54	70	51
**Gender**	M	M	M	M	M	M	M	M	F
**Ethnicity**	C	C	C	C	C	C	C	C	AA
**BP**	85	87	71	97	111	172	92	88	98
**HF etiology**	NI	I	I	NI	I	I	NI	I	NI
**EF (%)**	15–20%	10–15%	15–20%	10–15%	5–10%	5–10%	20–25%	10–15%	22–25%
**Diabetes**	–	+	–	–	+	–	–	+	–
**BNP (pg/mL)**	2810	2460	599	6260	7110	9960	991	1507	2550
**Creatinine (mg/dL**	1.51	1.56	2.07	1.29	1.81	1.55	0.96	1.58	1.03
**Beta blocker**	+	+	+	+	+	+	+	+	+
**ACE/ARB**	+		+	+		+	+	+	+
**Aldosterone antagonist**		+	+	+		+	+		+
**Diuretic**	+	+	+	+	+	+	+	+	+
**Statin**		+	+		+	+		+	
**Aspirin**	+	+	+	+	+	+	+	+	+
**Warfarin**		+			+	+	+		

M-male, F-female, C-Caucasian, AA-African-American, BP-Blood Pressure, HF-Heart Failure, NI-non Ischemic, I- Ischemic, EF- Ejection fraction, BNP- β-Natriuretic Peptide.

### DNA samples, genotyping and analysis

DNA samples were obtained from individuals (n = 855) from the Royal Brompton and Harefield NHS Trust Tissue Typing Service, UK, referred for cardiac transplantation due to dilated cardiomyopathy (DCM, n = 234) or ischemic cardiomyopathy (ICM, n = 621), and from individuals at the University of Minnesota (n = 152) referred for heart transplant or placement of a LVAD (DCM, n = 67 and ICM, n = 76) all according to institutional approved protocols. Primers as listed in the Table S4 were used to genotype samples for SNPs in miRNA binding sites on the commercially available high-throughput genotyping Sequenom MassARRAY platform (San Diego, CA, USA) as previously described [21]. SNPs were compared to that in a general population (Caucasian samples from 1000 Genome project, Phase 1 data, (http://www.1000genomes.org/). We identified 853 unique SNPs in 914 unique target sites in 763 unique 3′UTRs in dbSNP. From ESP5500 we identified 343 unique SNPs that were found in 348 unique target sites in 311 unique 3′UTRs (starting from an initial size of 14,024,295 SNPs, and 1,837,369 SNPs in the ESP5500 database). For details see Text S1 and Table S4.

### Cell culture and transfection

HEK 293T cells were purchased and maintained according to the manufacturer’s guidelines (ATCC). Transient expression of miR-16, -103, -107 or a scrambled control was achieved by using miRVana mimics according to the manufacturer’s instruction (Life Technologies). Lipofectamine 2000 was used according to the manufacturer’s protocol for transient transfection of HEK 293T cells (Invitrogen). Endpoints were analyzed 48 h post transfection.

### RNA isolation and Real Time Quantitative PCR (RTQPCR)

Total RNA was extracted with Trizol according to the manufacturer’s directions (Invitrogen). For miRNA, cDNA were synthesized by using the Taqman MicroRNA Reverse Transcription Kit (Applied Biosystems). For mRNA expression cDNA was synthesized by utilizing the SuperScript III kit (Invitrogen) [22]. Data is expressed relative to control and was calculated by the 2∧-deltadeltaCT method as described [22]. Taqman primers used in this study are listed in the Table S4.

### Dual Luciferase Reporter Assay

HEK 293T cells were co-transfected with either WT or mutated (harboring rs16958754) VPS4a 3′UTR reporter clones for miRNA target validation (Origene) and Renilla luciferase construct (Promega) with miR-16, -103, -107 or a scrambled control mimic according to the manufacturer’s protocol (Life Technologies). The 3′UTR of VPS4a was cloned downstream of the luciferase gene. An Internal ribosome entry site (IRES) is located upstream of luciferase. As the miR/RISC complex binds to the target, luciferase activity is reduced. If there is no interaction between the miR and the target, luciferase activity remains high. Luciferase activity was assessed using the Dual Luciferase Reporter Assay System (Promega) [23].

### Western Analysis

For details see Text S1.

### Cell number and Apoptosis

HEK 293T cells were plated and transfected with VPS4a cDNA plasmid (Origene) or an empty vector (Origene) using Lipofectamine 2000 according to the manufacturer’s instruction (Life Technologies). Increase in cells number was assessed every 24 h using a Hemocytometer. Apoptosis was assessed 48 h post transfection by staining with Hoechst 33342 for nuclear morphology, and the percentage of apoptotic nuclei was quantified as previously described [24].

### Statistics

A Student’s T-test was used for comparison of two groups and a One Way ANOVA with a post-hoc Tukey analysis was used for comparison of multiples groups. P<0.05 was considered significant. All values are presented as mean ± SE.

## Results

We identified 1175 SNPs in miRNA binding sites within the 3′ UTR of genes (Table S1). This supplemental table includes targets harboring the same SNP within the binding sites of multiple miRNA families. 137 genes contained a SNP in the 3′ UTR that was within the binding site of more than one miRNA family (Table S1).

**Figure 1:**
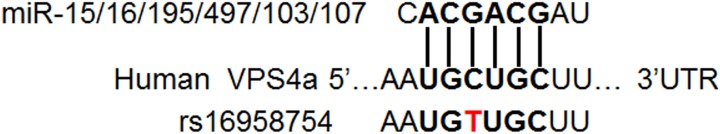
Predicted binding site of miR15/16/195/497/103/107 in 3′UTR of VPS4a. Binding site of miR15/16/195/497/103/107 and the SNP, rs16958754[C/T] in the 3′UTR of human VPS4a.

**Figure 2:**
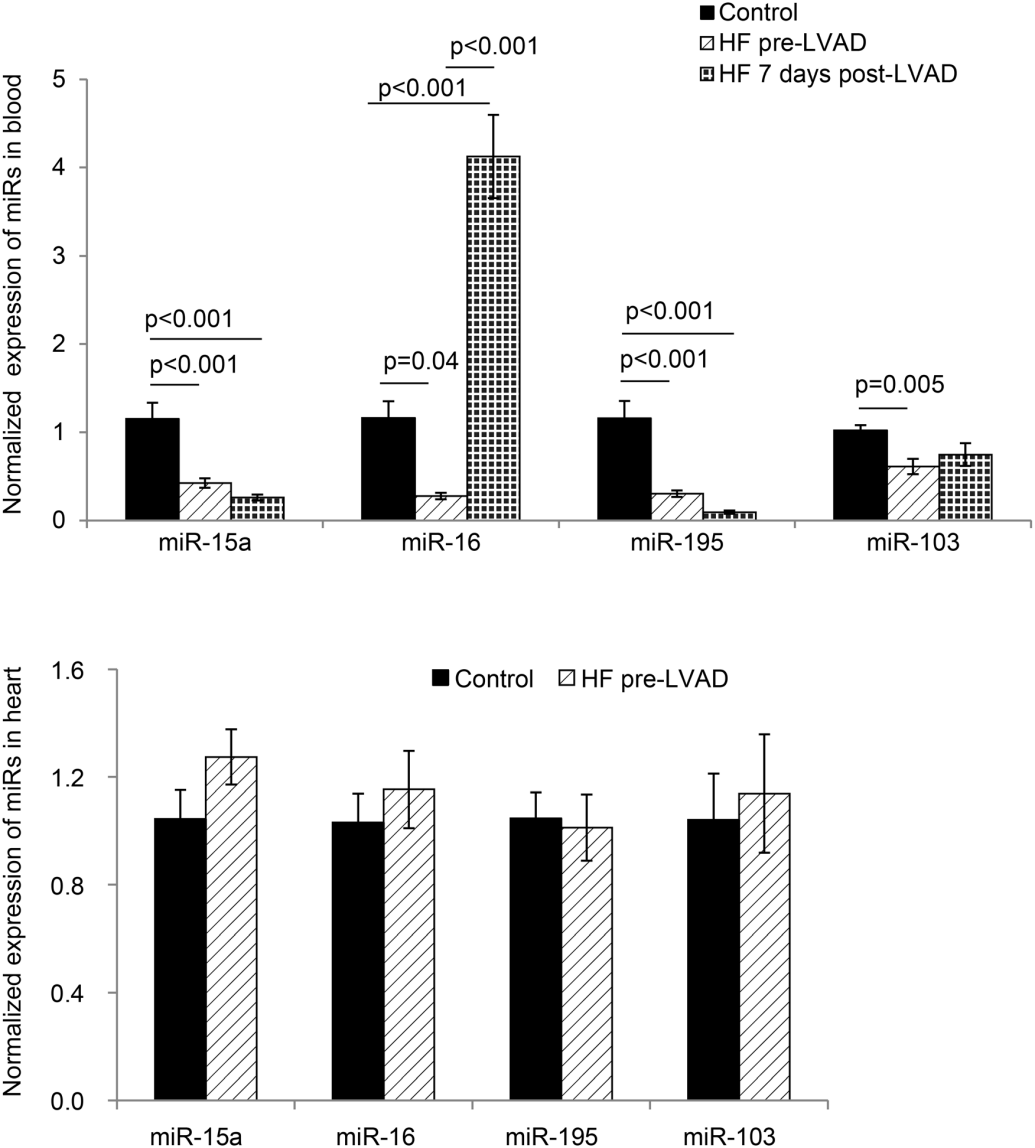
MiR-15a, -16, -195, and -103 expression in peripheral blood and heart tissue in patients with end-stage HF. (**A**) miR-15a, -16, -195, and -103 in peripheral blood were decreased in patients with end-stage HF prior to LVAD implant (pre-LVAD) (n = 9) compared to age, gender, and ethnicity matched healthy controls (n = 6). MiR-16 expression increased from end-stage HF prior to LVAD (HF pre-LVAD, n = 9) compared to patients with end-stage HF 7 days post LVAD (HF 7 days post-LVAD) (n = 9). MiR-15a, -16, -195, and -103 were decreased in patients with HF compared to controls. (**B**) Expression of miR-15/107 family in heart samples was not significantly different between non-failing controls (Controls) (n = 9) and end-stage HF samples (HF pre-LVAD) (n = 10). Samples were in duplicate and target expression normalized to SnU6 for A and B (mean ±SE).

24 SNPs were identified in binding sites specific for the miR-15 family. 11 SNPs were identified in binding sites specific for the miR-107 family. One SNP was shared between the miR-15 and the miR-107 family, rs16958754 [C/T] in VPS4a (Fig. 1). miR-15a, -16, -195 and -103 were decreased in peripheral blood samples of individuals with end-stage HF compared to controls (gender, age, and ethnicity matched) (p<0.02, Fig. 2A) (Table 1). MiR-16 in the peripheral blood was the only miRNA to increase 7 days post LVAD implantation (p<0.001, Fig. 2A). Heart tissue from end-stage HF patients (n = 10) showed no change in expression of miR-15a, -16, -195 and -103 compared to controls (n = 9, Fig. 2B).

**Figure 3:**
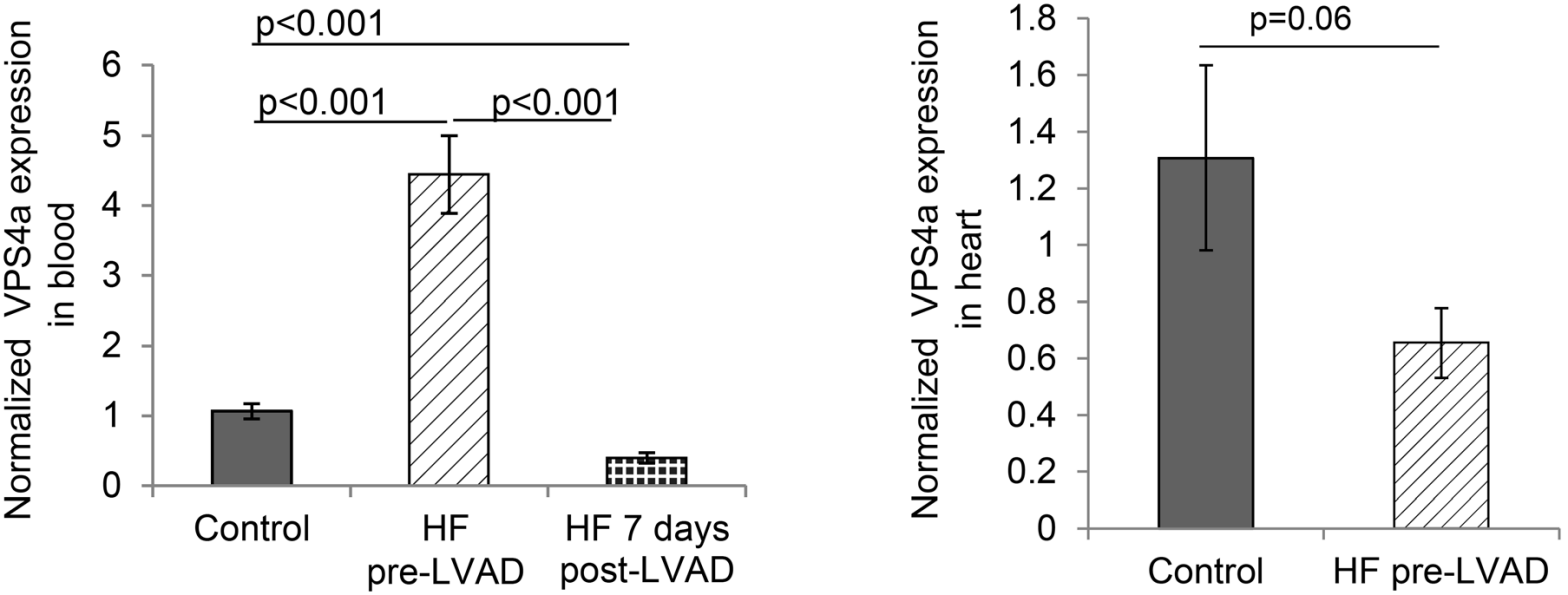
VPS4a expression in peripheral blood and heart tissue in patients with end-stage HF. (**A**) VPS4a transcript is higher in patients with end stage HF pre-LVAD (HF pre-LVAD) (n = 9) and is decreased 7 days post implant (HF 7 days post-LVAD) (n = 9) compared to age and gender matched controls (Control) (n = 6). (**B**) VPS4a transcript in heart was not different in patients with end stage HF (HF pre-LVAD, n = 10) compared to non-failing controls (n = 9). Samples were run in duplicate and target expression was normalized to 18S (mean ± SE).

VPS4a (the gene containing rs16958754 in the miR-15/107 binding site) was increased in peripheral blood from end-stage HF patients and decreased 7 days post-surgery as compared to controls (p = 0.0001) (Fig. 3A). VPS4a expression in heart tissue from HF patients was not different from non-failing controls (Fig. 3B).

**Figure 4:**
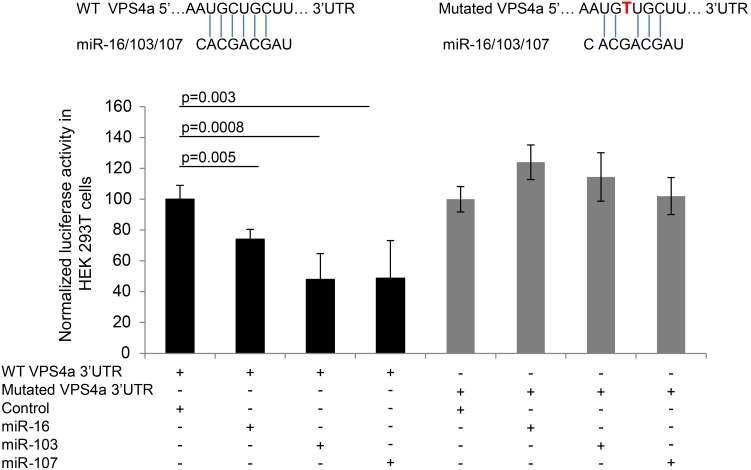
MiR-16, -103 and -107 interact with the 3′UTR of VPS4a. Binding of miR-16, -103 or -107 to WT 3′UTR of VPS4a decreases luciferase activity (n = 10) in HEK 293T cells. The SNP rs16958754[C/T] abolishes the interaction of miR-16, -103 or -107 to the mutated 3′UTR of VPS4a resulting in no change in luciferase activity (n = 10) (mean ± SE).

Studies in vitro showed that miR-16, miR-103 and miR107 inhibited luciferase activity of a 3′ UTR VPS4a reporter construct for mRNA target validation in HEK 293T cells (Fig. 4). Interaction between the 3′UTR VPS4a reporter construct and miR-16 was inhibited by the SNP, rs16958754 (Fig. 4). The endogenous levels of these miRs in HEK 293T cells exhibited crossing thresholds ranging from 23 (miR-16) to 27 (miR-107).

**Figure 5:**
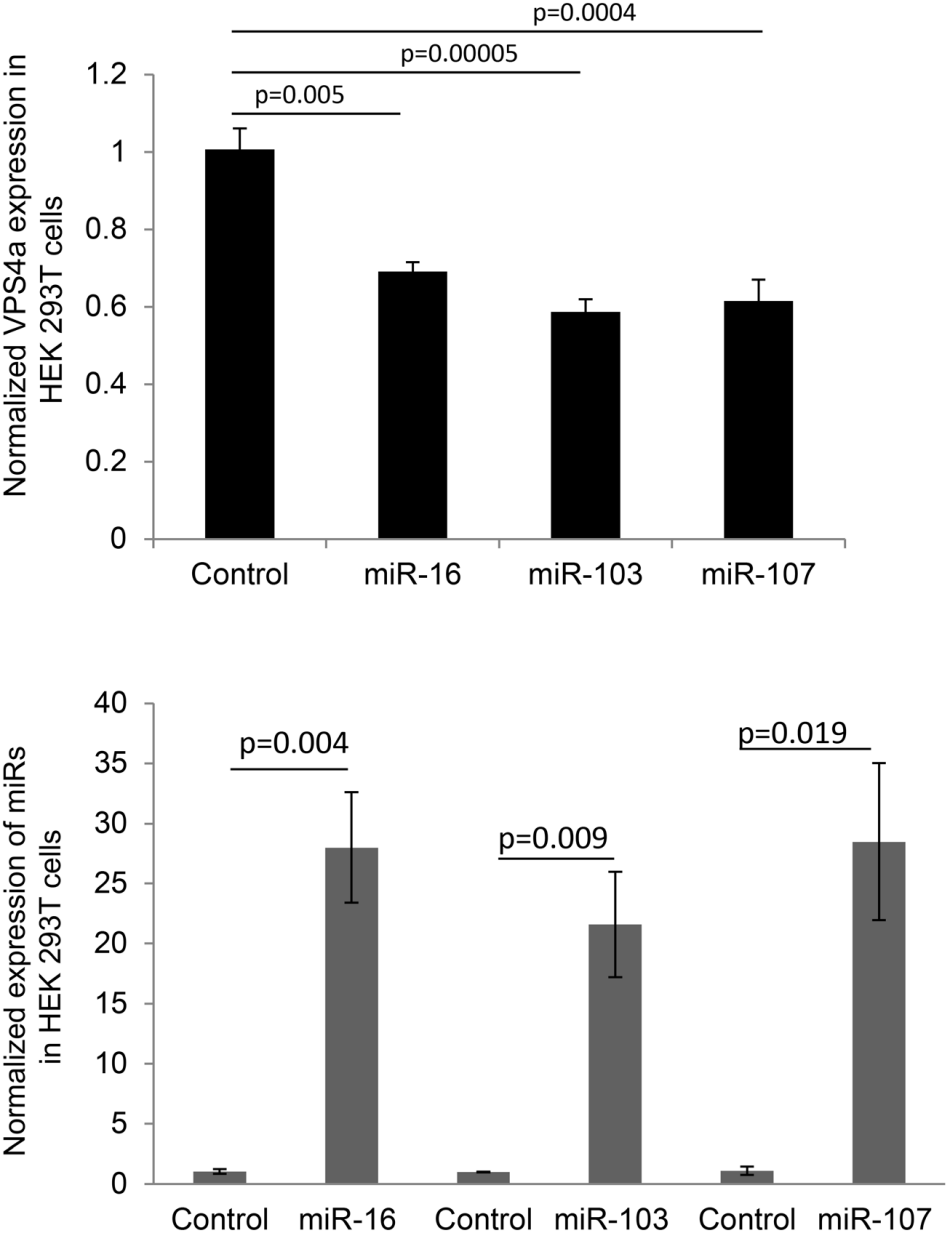
MiR-16, -103 and -107 decrease VPS4a mRNA expression. (**A**) Expression of VPS4a mRNA in HEK 293T cells decreases following overexpression of miR-16, or -103, or -107 (n = 3). Overexpression of a scrambled control mimic (control) had no effect (n = 3). Samples were run in duplicate and VPS4a expression normalized to 18S. (**B**) Overexpression of miR-16, -103 and -107 transcript *vs.* a scrambled control mimic (control) 48 h post transfection in HEK 293T cells (n = 3). Samples were run in duplicate and expression normalized to RNU38B (mean ± SE).

**Figure 6:**
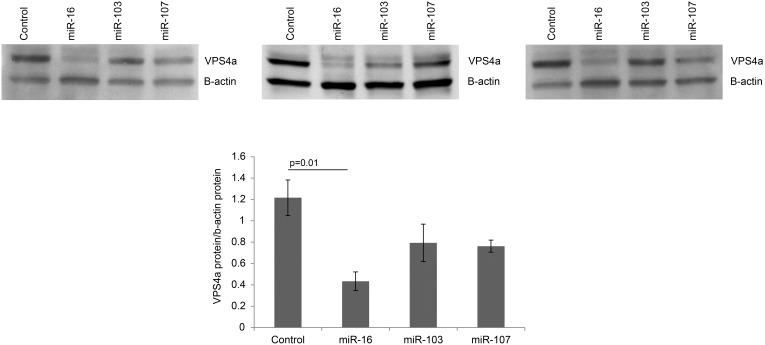
MiR-16 decreases protein expression of VPS4a. VPS4a protein is decreased following overexpression of miR-16. All 3 Western blots are shown along with densitometry. Overexpression of miR-103 and miR-107 or scrambled control had no significant effect. Bands were normalized to β-actin. Densitometry values are mean ± SE, n = 3.

**Figure 7:**
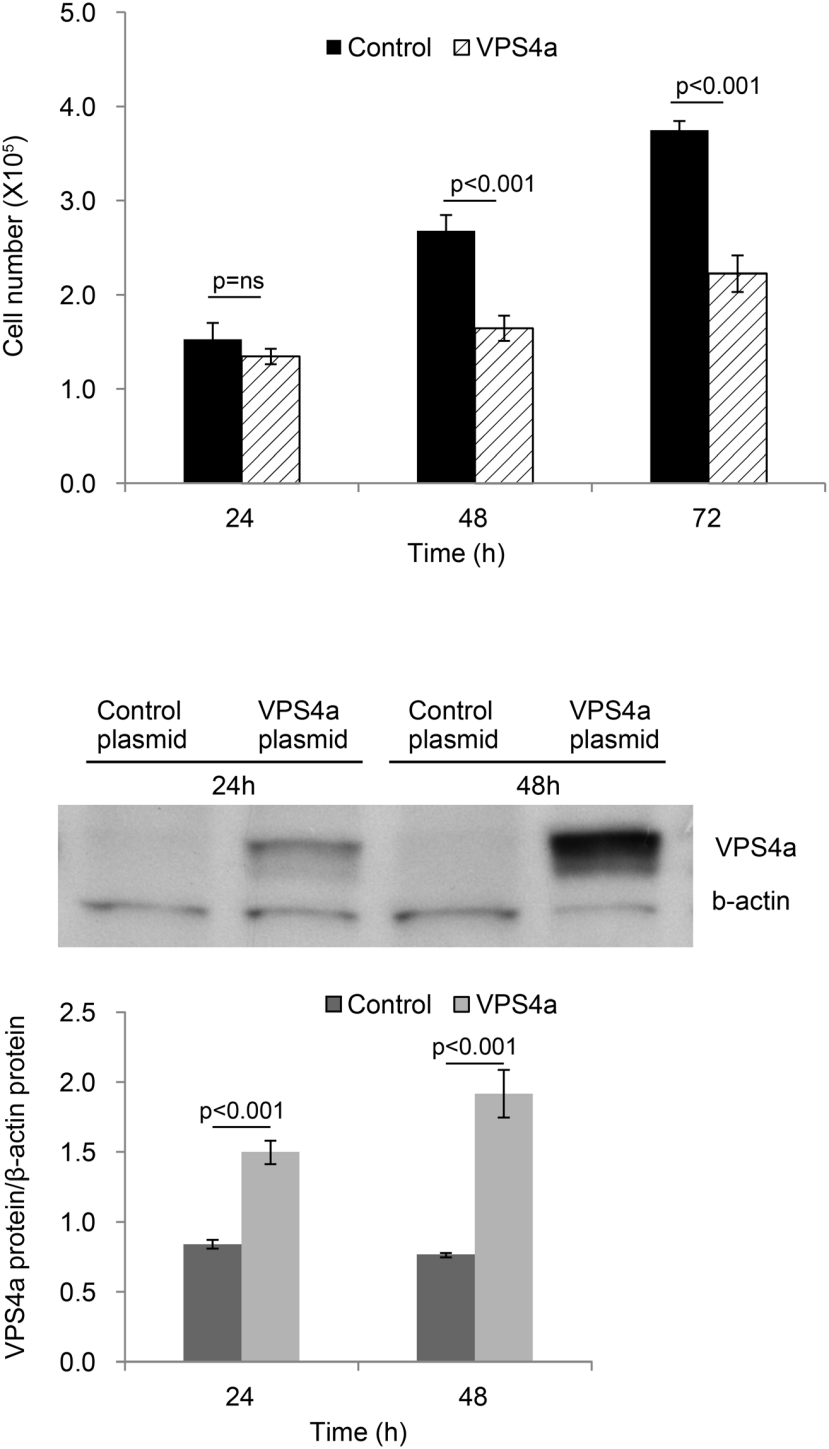
Overexpression of VPS4a decreases cell number. (**A**) Number of HEK 293T cells decreased following transfection with WT VPS4a compared to cells transfected with a control plasmid (Control). Values are mean ± SE, n = 4. (**B**) Western blot showing increased VPS4a protein following VPS4a transfection in HEK 293T cells *vs.* cells transfected with a control plasmid at 24 and 48 hours. Bands were normalized to β-actin. Densitometry values are mean ± SE, n = 3.

VPS4a mRNA was decreased following overexpression of miR-16, -103 and -107 (Fig. 5A) in HEK 293T cells. Figure 5B shows the overexpression levels of miR-16, -13, -107 transcripts compared to RNU38B, an internal control, 48 h post transfection. Only miR-16 decreased VPS4a protein abundance (Fig. 6). Cell number (HEK 293T) was decreased in response to up-regulation of VPS4a (Fig. 7A). No differences were noted in apoptosis in VPS4a overexpressing cells as compared to cells transfected with an empty vector (0.3±0.2% vs 0.3±0.4%, respectively). The level of VPS4a expression post transfection in HEK 293T cells is shown by Western blotting (Fig. 7B).

The two separate cohorts of end-stage HF samples were genotyped for a subset of the SNPs. The minor allele frequency (MAF) of these SNPs in the two HF populations did not differ significantly from the MAF published in dbSNP or 1000 genomes (Table S2). No SNP showed significant frequency difference between ethnic groups. Using data from the 1000 genomes project (http://www.1000genomes.org/), we were able to map 63 miRNA SNPs by their physical position on the genome. MAFs of these SNPs in Caucasians were compared between our samples and the 1000 genomes samples, and no significant difference was observed. Of interest, we observed several individuals carrying multiple alleles at multiple loci at low frequencies. Specifically, four individuals carry 4 rare alleles (at 4 loci) with frequency <1%, and nine individuals carry 3 rare alleles with frequency <1%. Assuming independence among the miRNA loci, we randomly permuted the rare alleles in our samples to estimate the expected number of individuals carrying multiple rare alleles (Table 2). The excess number of individuals than expected may suggest correlation or interaction between the miRNA loci surveyed in our study.

**Table 2:** Number of samples with multiple alleles from the two HF cohorts.

Number of Alleles	Number of samples	
	Observed	Expected*
**2**	15	9.3
**3**	9	0.4
**4**	4	0.1

The complete list of sample ID, SNPs, miRNAs, and gene targets with more than one SNP is given in the Table S3. *The expected numbers were calculated using 1000 permutations.

## Discussion

A major finding of this study is that 2–3 miRNA families may bind to a site in the 3′ UTR that contains a single SNP. One of these SNPs, rs16958754, in the gene, VPS4a, was identified in the binding region of both miR-15 and miR-107 families. These families share a common sequence, “AGCAGC”. miR-16, a member of the miR-15 family, is one of the top 10 highest expressed mi-RNAs in peripheral blood cells [12]. Expression of miR-16 in the blood was down-regulated in end-stage HF and up-regulated 7 days post unloading with a LVAD. In contrast, the target of miR-16, VPS4a, was up-regulated with HF, and down-regulated post LVAD in the blood (Fig. 3A). Studies *in vitro* validated that miR-16 decreased mRNA level of VPS4a. The presence of the SNP, rs26958754 abolished this effect. Although the presence of the SNP, rs16958754, interfered with miR-16 interaction with VPS4a, the MAF of this SNP in our two heart failure cohorts was rare (0.7 and 2.2%). The MAF of this SNP in normal controls in the 1000 genome project was 0. Taken together, these experiments suggest that miR-16 interacts with VPS4a and that circulating expression of miR-16 is down-regulated with HF while expression of its target, VPS4a, is increased. In response to LVAD therapy, circulating miR-16 is increased along with a concomitant decrease in VPS4a. In the rare case when a subject carries the SNP, rs16958754, it is possible that VPS4a levels have been high prior to HF given that miR-16 was elevated and not binding to the 3′UTR of VPS4a. It is speculated that VPS4a regulation through miR-16 would remain high during HF, given miR-16 is decreased and that miR-16 does not bind efficiently to VPS4a in patients with the SNP. Finally, it is possible that LVAD therapy would not decrease VPS4a through a miR-16 mediated mechanism in these subjects, given that miR-16 would increase with the LVAD yet not interact with its target VPS4a due to the SNP, rs16958754.

To define the cellular role of increased VPS4a in heart failure, we overexpressed VPS4a in HEK 293T cells and showed a significant decrease in cell number. This data agrees with the majority of studies showing that a decrease in miR-16 decreases cell number [25]–[27]. However not all studies show that miR-16 decreases cell number, which one might expect given miR-16 has multiple downstream targets [28]. VPS4a is thought to be part of an evolutionary ancient membrane remodeling system [19]. VPS4a is regulated by miRNAs 34a, 34c-5p, 449a, 449b, 574-3p, and -515-3p. To our knowledge, these miRNAs have not been reported to be altered in response to HF or LVAD therapy in humans. Furthermore, unlike miR-16, these miRNAs are not expressed at a high level in peripheral blood cells [12]. Expressions of many of the top miRNAs in peripheral blood are increased by 3–4 folds in platelets and reticulocytes including miR-16.

SNPs within miRNA binding sites are currently being used as prognostic and predictive cancer biomarkers [29]. The list of SNPs provided in this study may lead to future work in this direction in the area of cardiovascular disease as well as other diseases outside the realm of cardiovascular disease. The potential to utilize these SNPs and their targets as biomarkers for response to treatment are also future possibilities. We identified many targets with SNPs within a binding site that was recognized by multiple miRNAs. The potential for these SNPs and gene targets to be prognostic markers for blood diseases or immune related diseases in which T and B cells are critical looks to be very promising. This is particularly interesting given that many of the patients have 2 SNPs that share a common miRNA that is regulated in the context of cardiovascular disease including miR-129. The complete list of the sample reference numbers and SNPs, miRNAs, and gene targets with more than one SNP is given in the Table S3. In sum, this study shows that SNPs in miRNA binding sites, and/or levels of miRNA expression may impact the progression of heart failure and/or the response to treatment such as a LVAD.

The potential limitations of this study are that the HF cohorts we used to genotype were relatively small. We did genotype two cohorts (one from Minnesota and the other from London), both with end-stage HF. We have provided all of the SNPs identified in the miRNA binding sites for others to utilize. Secondly, the cell type we utilized for the modeling experiments were HEK 293T cells, an epithelial cell line derived from human embryonic kidney. Although experiments in primary cells may have been insightful, the goal was to test if VPS4a was indeed a downstream target of miR-16 and if the SNP interfered with the binding. For these experiments the HEK 293T cells provided clean, interpretable data.

In conclusion, the present study identified and validated VPS4a as a novel target of miR-16. The SNP rs16958754 [C/T] interfered with the binding of miR-16 to VPS4a. Loss of miR-16 in patients with end stage HF and the concomitant rise in VPS4a may lead to a significant decrease in circulating platelets and/or reticulocytes with end stage disease. In response to treatment with a LVAD, miR-16 increases and VPS4a decreases. This suggests a potential role for the miR-16-VPS4a signaling pathway in erythropoiesis.

## Supporting Information

Table S1 List of identified chromosomes, miRNA and SNPs with start and stop site.
(XLS)

Table S2 List of SNPs genotyped in this study.
(DOCX)

Table S3 Multiple rare alleles identified in individual with end-stage HF.
(DOCX)

Table S4 PCR and Extend Primer Sequences of 5 plexes and list of Taqman primers used for miRNA and mRNA expression by RTQPCR.
(DOCX)

Table S5 Samples utilized in the study.
(DOCX)

Text S1 **Expanded Methods.**
(DOCX)
