# Supplementary material for: Identification of a New Target of miR-16, Vacuolar Protein Sorting 4a
Source: PLoS One. 2014 Jul 17;9(7):e101509. doi: 10.1371/journal.pone.0101509 (PMC4102469; doi:10.1371/journal.pone.0101509)
Supplement: Table S2 — List of SNPs genotyped in this study. (DOCX) [file pone.0101509.s002.docx]

**Table S2. List of SNPs genotyped in this study. Position, SNPs genotyped in our samples, SNP name in 1000G (mapped by bp position), base pair position (hg19), GENE name, the miRNA predicted to bind to this region, the ancestral allele, the minor allele frequency (MAF) of both cohorts combined (missing if failed quality control in at least one cohort , MAF of samples in the UK (missing if failed quality control), MAF of MN cohort samples (missing if failed quality control), MAF in UK samples with reported Caucasian ancestry (missing if failed QC), and the MAF in 1000 genomes.**

| Chr | SNP | position | gene | miRNA | Allele | MAF | | | | |
| --- | --- | --- | --- | --- | --- | --- | --- | --- | --- | --- |
|  |  |  |  |  |  | All | UK | MN | White | 1000G |
| chr1 | 2334610 | 2334611 | RER1 | miR-24 | A | 0 | 0 | 0 | 0 |  |
| chr1 | rs114313009 | 7827903 | CAMTA1 | miR-129/129-5p | C | 0 | 0 | 0 | 0 |  |
| chr1 | 9427681 | 9427682 | SPSB1 | miR-24 | C | 0 | 0 | 0 | 0 |  |
| chr1 | 10479848 | 10479849 | PGD | miR-1/206 | C | 0 | 0 | 0 | 0 | 0.0026 |
| chr1 | 25893528 | 25893529 | LDLRAP1 | miR-133/miR-9 | C | 0 | 0 | 0 | 0 |  |
| chr1 | rs77861341 | 33329164 | FNDC5 | miR-129/129-5p | G | 0 | 0 | 0 | 0 |  |
| chr1 | rs118183123 | 36559030 | ADPRHL2 | miR-133 | C | 0 | 0 | 0 | 0 | 0 |
| chr1 | rs114408391 | 36770184 | THRAP3 | miR-133 | T | 0.004 | 0.004 | 0.007 | 0.002 | 0 |
| chr1 | rs1128665 | 36805312 | STK40 | miR-129/129-5p/miR-758/miR-134 | A | 0 | 0 | 0 | 0 |  |
| chr1 | rs77877901 | 45809609 | TOE1 | miR-129/129-5p | C | 0 | 0 | 0 | 0 |  |
| chr1 | rs115190046 | 151007824 | PRUNE | miR-485/485-5p/miR-221/222 | C | 0.001 | 0.001 | 0 | 0 | 0 |
| chr1 | rs6683364 | 151746202 | TDRKH | miR-485/485-5p | A | 0.142 | 0.145 | 0.125 | 0.154 | 0.1398 |
| chr1 | rs78605519 | 154930303 | PYGO2 | miR-202/202-3p/let-7/98 | C | 0.041 | 0.041 | 0.08 | 0.041 | 0.0303 |
| chr1 | rs116346657 | 156044213 | MEX3A | miR-129/129-5p | G | 0 | 0 |  | 0 |  |
| chr1 | rs75823770 | 156044214 | MEX3A | miR-129/129-5p | C | 0 | 0 |  | 0 |  |
| chr1 | rs115645935 | 156109094 | LMNA | miR-129/129-5p | C | 0 | 0 | 0 | 0 |  |
| chr1 | 156713233 | 156713234 | HDGF | miR-494 | T | 0 | 0 | 0 | 0 | 0 |
| chr1 | rs75390485 | 179072624 | ABL2 | miR-1/206 | G | 0 | 0 |  | 0 | 0 |
| chr1 | rs76046312 | 183596612 | ARPC5 | miR-133 | G | 0 | 0 | 0 | 0 |  |
| chr1 | rs11580973 | 210029185 | C1orf107 | miR-24 | A | 0.031 | 0.033 | 0.029 | 0.038 | 0.0237 |
| chr1 | rs114577052 | 225590146 | LBR | let-7/98/miR-202/202-3p | G | 0 | 0 | 0 | 0 | 0 |
| chr2 | rs75275902 | 43451047 | ZFP36L2 | miR-1/206 | C | 0.001 | 0 | 0.007 | 0 | 0 |
| chr2 | rs74621258 | 70053345 | ANXA4 | miR-1/206 | A | 0 | 0 | 0 | 0 |  |
| chr2 | rs75061955 | 80875430 | CTNNA2 | miR-129/129-5p | C | 0 | 0 | 0 | 0 |  |
| chr2 | rs79101693 | 89049874 | RPIA | miR-129/129-5p | A | 0.004 | 0.005 | 0 | 0.006 |  |
| chr2 | rs78891230 | 152695492 | CACNB4 | miR-129/129-5p | T | 0 | 0 | 0 | 0 |  |
| chr2 | 198368028 | 198368029 | HSPE1 | miR-1/206 | A | 0 | 0 | 0 | 0 |  |
| chr2 | rs77911328 | 208627843 | FZD5 | miR-24 | T | 0.045 | 0.042 | 0.065 | 0.051 | 0.0303 |
| chr2 | 238233394 | 238233395 | COL6A3 | miR-202/202-3p | A | 0 | 0 | 0 | 0 |  |
| chr3 | rs77484576 | 30046424 | RBMS3 | miR-129/129-5p | C | 0 | 0 | 0 | 0 |  |
| chr3 | rs115168088 | 30768469 | GADL1 | miR-1/206 | A | 0 | 0 | 0 | 0 | 0 |
| chr3 | 39184502 | 39184503 | AXUD1 | miR-15/16/195/424/497 | C | 0.003 | 0.004 | 0 | 0.005 |  |
| chr3 | rs114495432 | 45719721 | LIMD1 | miR-202/202-3p/let-7/98 | T | 0 | 0 | 0 | 0 |  |
| chr3 | rs56135778 | 57613454 | FAM116A | miR-494 | C | 0.18 | 0.185 | 0.146 | 0.176 | 0.1372 |
| chr3 | rs78606495 | 127788698 | SEC61A1 | miR-129/129-5p | A | 0 | 0 | 0 | 0 | 0 |
| chr3 | 149683947 | 149683948 | PFN2 | miR-485/485-5p | G | 0 | 0 | 0 | 0 |  |
| chr3 | rs74491004 | 185765587 | ETV5 | miR-129/129-5p | G | 0 | 0 | 0 | 0 |  |
| chr3 | rs116276176 | 194790088 | C3orf21 | miR-133 | G | 0 | 0 | 0 | 0 | 0 |
| chr3 | 197401836 | 197401837 | KIAA0226 | miR-24 | A | 0 | 0 | 0 | 0 | 0 |
| chr4 | 39553704 | 39553705 | C4orf34 | miR-133 | T | 0.002 | 0.002 | 0 | 0.003 | 0 |
| chr4 | rs114349450 | 47849831 | NFXL1 | miR-135/miR-24 | C | 0.003 | 0.003 | 0.007 | 0.002 | 0 |
| chr4 | rs1042279 | 111483133 | ENPEP | miR-133 | A | 0.055 | 0.063 | 0 | 0.059 | 0.0844 |
| chr4 | rs79590414 | 111538779 | PITX2 | miR-494 | G | 0 | 0 | 0 | 0 |  |
| chr5 | rs13357043 | 112349294 | DCP2 | miR-494 | A | 0.007 | 0.007 | 0.007 | 0.002 | 0 |
| chr5 | rs6892784 | 112349297 | DCP2 | miR-494 | C | 0.213 | 0.2 | 0.285 | 0.217 | 0.2045 |
| chr5 | rs17217562 | 141974653 | FGF1 | miR-133 | A | 0 | 0 | 0 | 0 | 0 |
| chr6 | rs116952349 | 32131040 | PPT2 | miR-15/16/195/424/497 | G | 0 | 0 | 0 | 0 |  |
| chr6 | rs115358880 | 32131049 | PPT2 | miR-15/16/195/424/497 | A | 0.002 | 0.002 | 0 | 0 | 0 |
| chr6 | rs75921733 | 70917184 | COL19A1 | miR-1/206 | G | 0.025 | 0.025 | 0.029 | 0.031 | 0.0132 |
| chr6 | rs75604548 | 87971645 | ZNF292 | miR-129/129-5p | A | 0.001 | 0.001 | 0 | 0.002 |  |
| chr6 | rs116138206 | 109484273 | C6orf182 | miR-494 | C | 0.001 | 0 | 0.007 | 0 | 0 |
| chr6 | rs79097024 | 119500583 | MAN1A1 | miR-129/129-5p | G | 0 | 0 | 0 | 0 |  |
| chr6 | rs116747379 | 146120444 | FBXO30 | miR-202/202-3p | C | 0.002 | 0.001 | 0.007 | 0.002 | 0 |
| chr7 | rs117139104 | 8126609 | GLCCI1 | miR-129/129-5p/miR-33/33ab | G | 0 | 0 | 0 | 0 |  |
| chr7 | 20180291 | 20180292 | 7A5 |  | G | 0.002 | 0.002 | 0 | 0 | 0 |
| chr7 | rs74332524 | 99632300 | ZKSCAN1 | miR-15/16/195/424/497/miR-103/107 | T | 0.024 | 0.021 | 0.043 | 0.025 | 0.0145 |
| chr7 | rs2718145 | 134654638 | CALD1 | miR-202/202-3p | T | 0.014 | 0.012 | 0.022 | 0.003 | 0.004 |
| chr7 | rs75037144 | 134654639 | CALD1 | miR-202/202-3p | A | 0.001 | 0 | 0.007 | 0 | 0 |
| chr7 | rs17168525 | 135613262 | MTPN | miR-181/let-7/98/miR-202/202-3p | A | 0.007 | 0.014 |  | 0.005 | 0.0119 |
| chr8 | rs114401825 | 1651465 | DLGAP2 | miR-129/129-5p/miR-33/33ab | C | 0 | 0 | 0 | 0 |  |
| chr8 | rs117054881 | 12941610 | DLC1 | let-7/98/miR-202/202-3p | T | 0 | 0 | 0 | 0 |  |
| chr8 | rs117033995 | 12943102 | DLC1 | miR-129/129-5p | G | 0 | 0 |  | 0 |  |
| chr8 | rs117411065 | 28203294 | ZNF395 | miR-129/129-5p | T | 0.002 | 0.002 | 0 | 0.003 |  |
| chr8 | rs28624549 | 37917639 | EIF4EBP1 | miR-202/202-3p | C | 0 | 0 | 0 | 0 |  |
| chr8 | rs77264178 | 66515086 | ARMC1 | miR-210 | A | 0.064 | 0.067 | 0.051 | 0.07 | 0.0594 |
| chr8 | rs78110727 | 105264087 | RIMS2 | miR-129/129-5p | C | 0 | 0 | 0 | 0 |  |
| chr8 | rs76675754 | 105264755 | RIMS2 | miR-129/129-5p | C | 0 | 0 | 0 | 0 |  |
| chr9 | rs75958174 | 3824580 | GLIS3 | miR-494 | A | 0.01 | 0.007 | 0.022 | 0.003 | 0.0013 |
| chr9 | rs115048919 | 91974080 | SECISBP2 | miR-24 | T | 0.005 | 0.005 | 0.007 | 0 | 0 |
| chr9 | rs116760928 | 100777863 | ANP32B | miR-1/206 | C | 0 | 0 | 0 | 0 |  |
| chr9 | rs114733331 | 100777868 | ANP32B | miR-1/206 | C | 0 | 0 | 0 | 0 |  |
| chr9 | rs10817559 | 116818033 | ZNF618 | miR-592/599/miR-129/129-5p | C | 0.241 | 0.246 | 0.21 | 0.258 | 0.1992 |
| chr10 | 102307935 | 102307936 | HIF1AN | miR-184 | T | 0.002 | 0.002 | 0 | 0.002 |  |
| chr11 | 18631404 | 18631405 | SPTY2D1 | miR-15/16/195/424/497 | T | 0.002 | 0.002 |  | 0 | 0 |
| chr11 | rs12286324 | 70315726 | SHANK2 | miR-494 | C | 0.001 | 0.001 | 0 | 0.002 | 0 |
| chr11 | rs78378706 | 70315729 | SHANK2 | miR-494 | G | 0.016 | 0.015 | 0.043 | 0.015 | 0.0145 |
| chr11 | rs59564714 | 73686038 | UCP2 | miR-15/16/195/424/497/miR-214/761 | A | 0.001 | 0.001 | 0 | 0 | 0 |
| chr11 | rs115588181 | 77930033 | GAB2 | miR-485/485-5p | A | 0.006 | 0.002 | 0.029 | 0.002 | 0 |
| chr11 | rs115149733 | 77930034 | GAB2 | miR-485/485-5p | T | 0.001 | 0.001 | 0 | 0.002 | 0 |
| chr12 | rs114937867 | 12048216 | ETV6 | miR-129/129-5p | C | 0 | 0 | 0 | 0 |  |
| chr12 | rs117478114 | 12048234 | ETV6 | miR-129/129-5p | A | 0.017 | 0.02 | 0 | 0.019 |  |
| chr12 | rs75401815 | 53807849 | SP1 | miR-1/206 | A | 0 | 0 | 0 | 0 | 0 |
| chr12 | 65110509 | 65110510 | GNS | miR-485/485-5p | T | 0.001 | 0 | 0.007 | 0 |  |
| chr12 | 69050993 | 69050994 | RAP1B/hCG_1757335 | miR-149/miR-149/miR-24/miR-24 | C |  |  | 0 |  | 0.0013 |
| chr12 | rs115544837 | 70209464 | RAB3IP | miR-129/129-5p | C | 0 | 0 | 0 | 0 |  |
| chr12 | rs117252134 | 79844330 | SYT1 | miR-1/206 | A | 0 | 0 | 0 | 0 | 0.0185 |
| chr12 | rs10187 | 108962804 | ISCU | miR-210 | T | 0.043 | 0.044 | 0.08 | 0.038 | 0.0383 |
| chr12 | rs75377774 | 111786650 | CUX2 | miR-133 | C | 0 | 0 | 0 | 0 |  |
| chr12 | 117583864 | 117583865 | FBXO21 | miR-15/16/195/424/497 | G | 0 | 0 | 0 | 0 |  |
| chr14 | rs76607827 | 45376362 | C14orf28 | let-7/98/miR-202/202-3p | T | 0 | 0 |  | 0 |  |
| chr14 | rs79446595 | 47310761 | MDGA2 | miR-494 | G | 0 | 0 | 0 | 0 |  |
| chr15 | rs77772971 | 31666796 | KLF13 | miR-1/206/miR-490/490-3p | C | 0.004 | 0.004 | 0.007 | 0 | 0 |
| chr15 | rs79303280 | 42059680 | MGA | let-7/98/miR-202/202-3p | C | 0.001 | 0.001 | 0 | 0 |  |
| chr15 | 52843993 | 52843994 | ARPP-19 | miR-15/16/195/424/497 | C | 0.027 | 0.029 | 0.036 | 0.035 | 0.0356 |
| chr15 | rs1666573 | 57840127 | CGNL1 | miR-15/16/195/424/497 | C | 0.218 | 0.213 | 0.25 | 0.223 | 0.2071 |
| chr15 | 99511674 | 99511675 | LOC145814 | miR-494 | A | 0 | 0 | 0 | 0 |  |
| chr16 | 9210798 | 9210799 | C16orf72 | miR-103/107/miR-15/16/195/424/497 | G | 0 | 0 | 0 | 0 |  |
| chr16 | 24226155 | 24226156 | PRKCB1 | miR-184 | G | 0 | 0 | 0 | 0 |  |
| chr16 | rs16958754 | 69358514 | VPS4A | miR-15/16/195/424/497/miR-103/107 | T | 0.009 | 0.007 | 0.022 | 0.002 | 0 |
| chr16 | rs111343596 | 70367607 | DDX19B | let-7/98/miR-202/202-3p/miR-196ab | A | 0 | 0 | 0 | 0 |  |
| chr16 | 74657799 | 74657800 | RFWD3 | miR-103/107/miR-15/16/195/424/497 | A | 0 | 0 | 0 | 0 |  |
| chr16 | rs4843173 | 86615269 | FOXL1 | miR-485/485-5p | T | 0.08 | 0.079 | 0.083 | 0.081 | 0.0673 |
| chr17 | 1946445 | 1946446 | DPH1/OVCA2 | miR-485/485-5p/miR-485/485-5p | G | 0 | 0 | 0 | 0 |  |
| chr17 | rs75817141 | 12044581 | MAP2K4 | miR-503/miR-15/16/195/424/497 | C | 0 | 0 | 0 | 0 | 0 |
| chr17 | rs115795511 | 40819965 | PLEKHH3 | miR-129/129-5p | T | 0.005 | 0.006 | 0 | 0.006 |  |
| chr17 | 41719240 | 41719241 | MEOX1 | miR-24 | A | 0.001 | 0.001 | 0 | 0.002 | 0.0013 |
| chr17 | rs75226959 | 42754857 | CCDC43 | miR-129/129-5p | G | 0 | 0 | 0 | 0 |  |
| chr17 | rs80136085 | 45699483 | NPEPPS | miR-129/129-5p | A | 0.001 | 0.001 | 0 | 0.002 |  |
| chr17 | rs75054818 | 47590405 | NGFR | miR-1/206/miR-299/299-3p | C | 0.015 | 0.017 | 0 | 0.019 |  |
| chr17 | rs80254118 | 57771918 | CLTC | miR-1/206 | C |  |  | 0 |  |  |
| chr17 | rs1051424 | 58024324 | RPS6KB1 | miR-129/129-5p | G | 0.207 | 0.209 | 0.197 | 0.212 | 0.1649 |
| chr18 | rs3087701 | 77915906 | PARD6G | miR-133 | G | 0.228 | 0.23 | 0.213 | 0.24 | 0.2045 |
| chr19 | rs74710244 | 47724112 | BBC3 | miR-494 | A | 0 | 0 | 0 | 0 | 0 |
| chr20 | rs115002326 | 9519752 | PAK7 | miR-129/129-5p | G | 0 | 0 | 0 | 0 |  |
| chr20 | rs77085264 | 31830941 | PLUNC | miR-15/16/195/424/497 | A | 0.002 | 0.002 | 0 | 0 | 0 |
| chr20 | rs117820837 | 54943386 | C20orf108 | miR-448/miR-153/miR-129/129-5p | A | 0 | 0 | 0 | 0 |  |
| chr21 | rs77613170 | 17250882 | USP25 | miR-15/16/195/424/497/miR-503 | A | 0 | 0 | 0 | 0 | 0.0079 |
| chr21 | rs12140 | 28209395 | ADAMTS1 | miR-503/miR-15/16/195/424/497 | G | 0.063 | 0.059 | 0.167 | 0.067 | 0.0712 |
| chr21 | rs78268613 | 28209397 | ADAMTS1 | miR-503/miR-15/16/195/424/497 | G | 0 | 0 | 0 | 0 |  |
| chr21 | 33040892 | 33040893 | SOD1 | miR-1/206 | C | 0 | 0 | 0 | 0 | 0 |
| chr22 | rs117913906 | 17599448 | CECR6 | miR-129/129-5p | T | 0 | 0 | 0 | 0 | 0 |
| chr22 | rs117617596 | 36140130 | RBM9 | miR-129/129-5p | G | 0.019 | 0.02 | 0.029 | 0.043 | 0.0132 |
| chr22 | rs116654628 | 40806518 | MKL1 | miR-1/206 | G | 0 | 0 | 0 | 0 | 0 |
