# Supplementary material for: Identification of a New Target of miR-16, Vacuolar Protein Sorting 4a
Source: PLoS One. 2014 Jul 17;9(7):e101509. doi: 10.1371/journal.pone.0101509 (PMC4102469; doi:10.1371/journal.pone.0101509)
Supplement: Table S3 — Multiple rare alleles identified in individual with end-stage HF. (DOCX) [file pone.0101509.s003.docx]

| Table S3. Multiple rare alleles identified in individual with end-stage HF. | | | | |
| --- | --- | --- | --- | --- |
| Patient ID | SNP | miRNAfamily | Gene | MAF |
| G4023 | 18631404 | miR-15/16/195/424/497 | spty2d1 | 0.001 |
| G3925 | 20180291 | miR-494 | 7a5 | 0.001 |
| G4723 | 20180291 | miR-494 | 7a5 | 0.001 |
| G3932 | rs114349450 | mir24 | nfxl1 | 0.002 |
| G4505 | rs114349450 | mir24 | nfxl1 | 0.002 |
| 11112 | rs114349450 | mir24 | nfxl1 | 0.002 |
| G4023 | rs114408391 | mir133 | thrap3 | 0.002 |
| G5465 | rs114408391 | mir133 | thrap3 | 0.002 |
| 11114 | rs114408391 | mir133 | thrap3 | 0.002 |
| A2011-05_31 | rs115048919 | mir24 | secisbp2 | 0.003 |
| G2864 | rs115048919 | mir24 | secisbp2 | 0.003 |
| G3135 | rs115048919 | mir24 | secisbp2 | 0.003 |
| G3153 | rs115048919 | mir24 | secisbp2 | 0.003 |
| G4723 | rs115048919 | mir24 | secisbp2 | 0.003 |
| G4455 | rs115190046 | mir485 | prune | 0.001 |
| G4593 | rs115358880 | mir15/16/19 | ppt2 | 0.001 |
| A2010-027_25 | rs115588181 | mir485 | gab2 | 0.003 |
| G4593 | rs115588181 | mir485 | gab2 | 0.003 |
| 11114 | rs115588181 | mir485 | gab2 | 0.003 |
| A0222 | rs115795511 | mir129 | plekhh3 | 0.003 |
| G3060 | rs115795511 | mir129 | plekhh3 | 0.003 |
| A2010-027_25 | rs116138206 | mir494 | c6orf182 | 0.001 |
| A2010-027_25 | rs116747379 | mir202 | fbx030 | 0.001 |
| G3368 | rs116747379 | mir202 | fbx030 | 0.001 |
| G2633 | rs117411065 | mir129 | znf395 | 0.001 |
| G2670 | rs117411065 | mir129 | znf395 | 0.001 |
| G3458 | rs117478114 | mir129 | etsv6 | 0.009 |
| G3932 | rs12286324 | mir494 | shank2 | 0.001 |
| A2011-04_30 | rs13357043 | mir494 | dcp2 | 0.004 |
| G2878 | rs13357043 | mir494 | dcp2 | 0.004 |
| G3153 | rs13357043 | mir494 | dcp2 | 0.004 |
| G4455 | rs13357043 | mir494 | dcp2 | 0.004 |
| G4744 | rs13357043 | mir494 | dcp2 | 0.004 |
| A2011-05_31 | rs16958754 | mir103/107 | vps4A | 0.005 |
| 11111 | rs16958754 | mir103/107 | vps4A | 0.005 |
| G3153 | rs16958754 | mir103/107 | vps4A | 0.005 |
| G3925 | rs16958754 | mir103/107 | vps4A | 0.005 |
| G4040 | rs16958754 | mir103/107 | vps4A | 0.005 |
| G4505 | rs16958754 | mir103/107 | vps4A | 0.005 |
| G4744 | rs16958754 | mir103/107 | vps4A | 0.005 |
| 11112 | rs16958754 | mir103/107 | vps4A | 0.005 |
| G3135 | rs17168525 | let7/98 | mtpn | 0.007 |
| G3458 | rs17168525 | let7/98 | mtpn | 0.007 |
| *11113 | rs17168525 | let7/98 | mtpn | 0.007 |
| A2011-05_31 | rs2718145 | mir202 | cald1 | 0.007 |
| G2864 | rs2718145 | mir202 | cald1 | 0.007 |
| G2878 | rs2718145 | mir202 | cald1 | 0.007 |
| G3368 | rs2718145 | mir202 | cald1 | 0.007 |
| G4455 | rs2718145 | mir202 | cald1 | 0.007 |
| G4505 | rs2718145 | mir202 | cald1 | 0.007 |
| G4593 | rs2718145 | mir202 | cald1 | 0.007 |
| G4723 | rs2718145 | mir202 | cald1 | 0.007 |
| G5465 | rs2718145 | mir202 | cald1 | 0.007 |
| G4744 | rs59564714 | mir214 | ucp2 | 0.001 |
| G4040 | rs75054818 | mir299 | ngfr | 0.007 |
| 11112 | rs75275902 | mir1/206 | zfp36l2 | 0.001 |
| A2010-027_25 | rs75958174 | mir494 | glis3 | 0.005 |
| A2011-04_30 | rs75958174 | mir494 | glis3 | 0.005 |
| 11111 | rs75958174 | mir494 | glis3 | 0.005 |
| G1938 | rs75958174 | mir494 | glis3 | 0.005 |
| G2864 | rs75958174 | mir494 | glis3 | 0.005 |
| G4723 | rs75958174 | mir494 | glis3 | 0.005 |
| G4744 | rs75958174 | mir494 | glis3 | 0.005 |
| G5465 | rs75958174 | mir494 | glis3 | 0.005 |
| G1938 | rs77085264 | mir15/16/19 | plunc | 0.001 |
| G4593 | rs77085264 | mir15/16/19 | plunc | 0.001 |
| G3135 | rs77772971 | mir490 | klf13 | 0.002 |
| G4023 | rs77772971 | mir490 | klf13 | 0.002 |
| A0222 | rs79101693 | mir129 | rpia | 0.002 |
| G2633 | rs79101693 | mir129 | rpia | 0.002 |
| G2670 | rs79101693 | mir129 | rpia | 0.002 |
| G3060 | rs79101693 | mir129 | rpia | 0.002 |

*Note that "11113" carries a homozygote of two rare alleles, so only appears once in the list
