# Supplementary material for: Identification of a New Target of miR-16, Vacuolar Protein Sorting 4a
Source: PLoS One. 2014 Jul 17;9(7):e101509. doi: 10.1371/journal.pone.0101509 (PMC4102469; doi:10.1371/journal.pone.0101509)
Supplement: Table S4 — PCR and Extend Primer Sequences of 5 plexes and list of Taqman primers used for miRNA and mRNA expression by RTQPCR. (DOCX) [file pone.0101509.s004.docx]

**Table S4:** PCR and Extend Primer Sequences of 5 plexes and the list of Taqman primers used for miRNA and mRNA expression by RTQPCR.

| **SNP_ID** | **1st-PCR PRIMER** | **2nd-PCR PRIMER** | **EXTEND PRIMER** |
| --- | --- | --- | --- |
| rs77613170 | ACGTTGGATGTTCCTGTCACAGGGTTTGCT | ACGTTGGATGGTCATTTTGTCTTTTGCAG | TTGCTTGTTGCTGCT |
| rs12140 | ACGTTGGATGGGATCTTTGACCAGCACTGT | ACGTTGGATGGAAGTTTTAATTCTCGGTAG | ACTGTTTATGGCTGCT |
| rs76675754 | ACGTTGGATGTTGCATGGACACAGATTAGC | ACGTTGGATGAAATTGTTGAAAATGGCTG | gTGGGGCTGCAACTTG |
| rs116654628 | ACGTTGGATGAGGAGCTCTCCACCCACTC | ACGTTGGATGCGAAGCTCCTCCTGTCCTA | aCCACCCACTCACATTC |
| rs115048919 | ACGTTGGATGCCTGCTTTCTGCAAACTCAC | ACGTTGGATGGAATACTTTGGCTGCTAAGG | GCAAACTCACTCACTTG |
| rs115358880 | ACGTTGGATGCACCCATACAGCCAGATACG | ACGTTGGATGGAAGTTGGGAAGTGAGAAAC | gGCCAGATACGGAGGAG |
| rs79590414 | ACGTTGGATGTATACTAAATGCCACACTG | ACGTTGGATGATTGCATCCACCAGAGAAAC | cTGCCACACTGTATGTTT |
| rs75037144 | ACGTTGGATGGGTAGGAATTGAGACTAACAC | ACGTTGGATGGCTGGATACATACAGTGGAG | TTTGGTTAAGTCCACTGA |
| rs115588181 | ACGTTGGATGATCTGGTAGAGAGTCAGGTC | ACGTTGGATGCCACCTTAGTTAGGAGCTAC | TCTAAAGGACAGGAAGAG |
| rs56135778 | ACGTTGGATGGATGCTTATGTAGTGCCTTC | ACGTTGGATGGAAAAGCTTCAAGACTAGCAG | TCTGTTGTCCTACATGTTT |
| rs13357043 | ACGTTGGATGGTGTTTTAAAGAAATGCAGGG | ACGTTGGATGACTCGTGTTTCTTTCTACTC | gGAAATGCAGGGAGGCAAT |
| rs114577052 | ACGTTGGATGGGCAAACAAACACCTGCAAT | ACGTTGGATGGAAGGGTATTTTTCTTGATCC | ccACACCTGCAATTGAGGTA |
| rs10187 | ACGTTGGATGCTTAGATGTTCAGAAGCCGC | ACGTTGGATGAGAGTCATAACGTGAACAGC | CACTGAAGAGCTATGAGATA |
| rs11580973 | ACGTTGGATGGGCTGATTTAGGAGTTGTGC | ACGTTGGATGCAAAAGGTAGACAGCTTTGG | cctgaCCATCTGTTCTCCCAG |
| rs117478114 | ACGTTGGATGTATTTGCAACTCACTGGCCC | ACGTTGGATGGTGGGGTTTATAAGGGACTG | TGAGAAAAGGCATTTTTTTTT |
| rs75226959 | ACGTTGGATGGTTACCTCTTCATACACTCC | ACGTTGGATGGTCAGAAGACAGCAATTTA | caccCCTATAACTGTGGCATTG |
| rs78606495 | ACGTTGGATGTTTGCTTTTTATCCTGGCAC | ACGTTGGATGTTCCCACTTCTCTGGCAGTC | cttgTATCCTGGCACTGGCAAA |
| rs117033995 | ACGTTGGATGGGGTCTGTATTCATAGACCT | ACGTTGGATGTGCTTCTCAGAAGCAATTTG | tgCTGGAATATGTAAGACTGTG |
| rs78378706 | ACGTTGGATGATGCGAACACGTGTTGTTAC | ACGTTGGATGGGCCACAATATTGATTTTCCC | TTACACAAACTATAGTGTGAAAC |
| rs114733331 | ACGTTGGATGGGTGGTAGATTACCAAGAGG | ACGTTGGATGCCCAAAGAGCCAAAGAATAG | gaggtACTACATGGAAGGAAGGC |
| rs75390485 | ACGTTGGATGTAAAAAACCTAGTTAGTCC | ACGTTGGATGGTCATTAAAATAATGTGAAC | gaacACCTAGTTAGTCCTACATTC |
| rs3087701 | ACGTTGGATGTACATTTATACTTTGAGAAC | ACGTTGGATGGCTCCGACAACCTAGAAAAA | atttcTACTTTGAGAACTCTGACC |
| rs117054881 | ACGTTGGATGGAGGTACAAAACCCAAAAGAA | ACGTTGGATGCAAACATAGATAAAGCATGA | AAACCCAAAAGAATATCTGAGGTA |
| rs116747379 | ACGTTGGATGCAATACGCCCAATTCAAACC | ACGTTGGATGCTCCTGGTATTATATATTTGC | gaCCAATTCAAACCTAAATGAGGTA |
| 10479848 | ACGTTGGATGAAAATAGGGCAAAGGGCCAG | ACGTTGGATGAATGCCTGATCATGCTGCTC | aacctTGCCATGAGGCACATGGAAT |
| rs77911328 | ACGTTGGATGTTGATACGGGTTTGGTGCAG | ACGTTGGATGGGACTTCAACTACTGACTCG | gggtTGCAGGTGAATTTATTACTGA |
| rs16958754 | ACGTTGGATGTTTTCCATCTTTTGTTCCCC | ACGTTGGATGCACTTCCAGGTGATTTTATC | cccgTTGTTCCCCTAAATTAATGCTG |
| rs77877901 | ACGTTGGATGAAGTCTGAAAATGTCTTGGG | ACGTTGGATGAACCAAAGATGAAGCTGGTC | gagtAAAATGTCTTGGGAAAGTTTTA |
| rs115190046 | ACGTTGGATGTGGGTCAACACGCCATGAAC | ACGTTGGATGTCTGTGTGGTTGTAGCAAGG | ggttAACCACACCCCTATTCGTGCTAC |
| rs79303280 | ACGTTGGATGGCTGACCCTGGTATAAGAAG | ACGTTGGATGCTTCTTAGAGAGGATGAGGG | tcccgTCTGATCATGTTAAAATGTGTT |
| rs75958174 | ACGTTGGATGAAGGACTTCATGAACAAGAC | ACGTTGGATGGTTTTGTGATTTGTCACAG | AACAAGACAAAATAACTTATGATTATA |
| 39184502 | ACGTTGGATGACTGTTACGCAGACTCTGGG | ACGTTGGATGACCAGGATGTCTTTTCCCAG | aggttGGGAGCCCCATAATTACAAGAAA |
| rs75275902 | ACGTTGGATGAAAATCAACCCAGCACCAGC | ACGTTGGATGTCCCACATCTGGACTTAAC | ACCAACCACCATTCC |
| rs75054818 | ACGTTGGATGGGTTGGCTGGAGCATCGGT | ACGTTGGATGAGTGAGTCCACTGCCACATC | AGCATCGGTTGTCGG |
| rs4843173 | ACGTTGGATGGCTGTCACTGGATATTTTGC | ACGTTGGATGCAGTCACTGAGAGGTTAATG | ATCCCAAACTCTCAGC |
| rs1666573 | ACGTTGGATGTTACAAACCAGCCACCCTAC | ACGTTGGATGAACCAGACCTTCTCACGAAC | CACATTCCAAACCCAAC |
| rs78110727 | ACGTTGGATGAAATTGTTGTCACAGCAACC | ACGTTGGATGAAGCATGCAGTGTTACCAGG | tcCAGCAACCAGCGTTA |
| rs115168088 | ACGTTGGATGCACAATGAAGTCCAGGGTTG | ACGTTGGATGGCTGAATCCCAGATTCTGTA | TCCAGGGTTGTACAACA |
| rs74332524 | ACGTTGGATGGAAAACAAAAACGACATTGG | ACGTTGGATGGGTAGGCTACCAGTGATCAT | AACGACATTGGGACATG |
| rs59564714 | ACGTTGGATGTAGAGACAAAGCCAGAGGTG | ACGTTGGATGTCATGGCTGCCTGCACTTC | gaAGAGGTGATCAGGTCA |
| rs77861341 | ACGTTGGATGGTCCTAGTCAGATGAAGGAG | ACGTTGGATGGGCCAGGCCTTTTAGTTTTG | ACTACAAACCCAAATTCTG |
| rs117252134 | ACGTTGGATGGACACTATTATGAAGCTAC | ACGTTGGATGACACGGCAGTCACAGTGTTA | TGAAGCTACTAGTCATTCC |
| rs76607827 | ACGTTGGATGTAATCCTCACAACAACCCTG | ACGTTGGATGCAGGAAATGCACTTTATTTGG | gggcACAACCCTGTGAGGT |
| rs78891230 | ACGTTGGATGGGGCTGCTGTCATTTTATGC | ACGTTGGATGATCCATCTAGACTCAAGGGC | ggggATGCTAAGTAAGGGGC |
| rs117913906 | ACGTTGGATGGCCATGGCAAATGCAACTTC | ACGTTGGATGTGCATCATGCACTGATGCTG | CTTCTGAGTATTTTGTCTTCA |
| 156713233 | ACGTTGGATGACACCATTAACCCTTCTCCC | ACGTTGGATGAGACAGCAGCAGGAACAGG | ggtacCAGCCTGTAGCCACCA |
| rs111343596 | ACGTTGGATGTACCATCCCCCATCATTTGC | ACGTTGGATGACAACGGCACAAGTAGAGAG | CAAACATAATTTGAAGTGAGG |
| rs75061955 | ACGTTGGATGCTGGAAGTGTAATGGTAGAAC | ACGTTGGATGTCAATTCATGGGCCACACAC | agggATTGCTTCTGTTTCAGTG |
| rs17217562 | ACGTTGGATGCTTTGCAGAGCCTTTTACCC | ACGTTGGATGACTGGCCAGCCAGTTGACTC | gaaggTTAGAACAGAGGGACCA |
| rs114349450 | ACGTTGGATGGAAATATTTCTCTTACTGC | ACGTTGGATGTTAATAATTCAAACCAACAC | ATATTTCTCTTACTGCAGATATG |
| rs80136085 | ACGTTGGATGTTGAAAAAGGAAAATCAGC | ACGTTGGATGTTCACAGTTTCGTTATGCTC | ggggAGGAAAATCAGCAATTCAG |
| rs114495432 | ACGTTGGATGCCCTTCACTTTCAGTCTTCC | ACGTTGGATGTCTGAGTCCAGTGTGAAAGC | ccctAGTCTTCCACACACAAAAAA |
| rs116138206 | ACGTTGGATGCCTTGAATTGTCTAAAGTGG | ACGTTGGATGCTGCAACTTCATGTTTTATAG | ATAACTTTGTGACATTTTGAATCA |
| rs75921733 | ACGTTGGATGATCATAGCAATTGTAAAGC | ACGTTGGATGCTATAGCAATTGCAAATCAGC | ATAGCAATTGTAAAGCAAAACATA |
| rs12286324 | ACGTTGGATGCACGTGTTGTTACACAAACT | ACGTTGGATGCCACAATATTGATTTTCCC | ggtgGTTACACAAACTATAGTGTGA |
| 238233394 | ACGTTGGATGTGGCTGACTCCTTCTTCTTC | ACGTTGGATGGTCATCAGTGTGATGGGAAC | cgcttACTCCTTCTTCTTCAAGAGGT |
| rs79446595 | ACGTTGGATGTTTGTTGGCAGGCCATTAGG | ACGTTGGATGAAGCACAAAGTCATGGCTGG | cattGTAACAAGCAAACTATTCATTT |
| rs6892784 | ACGTTGGATGCAGTGCAAACTCGTGTTTC | ACGTTGGATGTTAAAGAAATGCAGGGAGGC | aTCTTATAAACAGAAAATGTCTTCAG |
| rs2718145 | ACGTTGGATGGCTGGATACATACAGTGGAG | ACGTTGGATGGGTAGGAATTGAGACTAACAC | gggtAGTGGAGTTCTATAAACTCATA |
| rs74491004 | ACGTTGGATGCTGATTTTTGTAGTTTTGG | ACGTTGGATGAGGACACAGTGCACAGTTAC | ttGTATTTAAAGCAAAAAAACGAAAAA |
| rs116346657 | ACGTTGGATGCAACACAGAGGAGAGAAAAC | ACGTTGGATGTGGTAATAAACGCCTTTAT | aaaACAGAGGAGAGAAAACAAAAATAG |
| rs1042279 | ACGTTGGATGGTTTAAATACCAGTACTTG | ACGTTGGATGTTAGGTCTGTATAATTCTG | ccttcTTAAATACCAGTACTTGAGGGAC |
| rs116760928 | ACGTTGGATGCCCAAAGAGCCAAAGAATAG | ACGTTGGATGGGTGGTAGATTACCAAGAGG | ttttgGAGCCAAAGAATAGTTCCTGTGA |
| rs75817141 | ACGTTGGATGTACGTCTTGCTTCCTCTCAG | ACGTTGGATGATCAAATGCCAGCTACTCCC | gattgTTTCTAGAGTCTGATGTAGCAGC |
| 102307935 | ACGTTGGATGTGTCCTCATCAATGAAGCGG | ACGTTGGATGTGATCAAGGGCCGATACAAC | TGAAGCGGTGTGGAC |
| 74657799 | ACGTTGGATGAGGCGCTAGCAAACAACATC | ACGTTGGATGGGTCCACATCTATAAGTGGG | ACATCTAACCAGCAGC |
| rs80254118 | ACGTTGGATGTGTTGTGCTGTATCCTGTGC | ACGTTGGATGCACACAAGATTGGAATCATGG | GCTTTTTCTGTGGGAC |
| rs116952349 | ACGTTGGATGCCACCCATACAGCCAGATAC | ACGTTGGATGGAAGTTGGGAAGTGAGAAAC | GGAGGAGCAGCAGCAG |
| rs28624549 | ACGTTGGATGTTTCTCCCTCACTCAGGGCA | ACGTTGGATGTCCTGCATCAGTGGAGGC | TGAACACCAGCAGATAC |
| 1946445 | ACGTTGGATGTTAACTGGTTCTGGTGAGGC | ACGTTGGATGTCCTACATCCAGCTCCTCTA | GGCATGGATGACGGAGG |
| 197401836 | ACGTTGGATGTGAAGAAAGCACGTGCAGCC | ACGTTGGATGGGGCGAGTCCTTCAAAGAG | aCACACCTGTTGCAGAAC |
| rs78605519 | ACGTTGGATGTGACAGGGCCTCACTTGGAT | ACGTTGGATGAAGGAAAGCTTGAAAAGGCG | CATGGAGGAAATGAGGTA |
| rs17168525 | ACGTTGGATGCTGAGTATCAGTCCCTAACC | ACGTTGGATGTATTAAGACCAAGTCATGC | CCTCCCCTAAAAATTTGAG |
| rs77264178 | ACGTTGGATGCATGTGTTTGGTTTCTGATTC | ACGTTGGATGGCCCTGAAAGTTATTGTTGC | GTTTCTGATTCAAGTGACA |
| rs118183123 | ACGTTGGATGGCAGGGTTTCTGATTGGAGC | ACGTTGGATGGAAGAGTTGATGAGGGCTAC | ggTGATTGGAGCTGTAGTT |
| rs117820837 | ACGTTGGATGCAGTCTCCCATTTATGTCCC | ACGTTGGATGAACCTGTAGGTTTCACCATC | ttTCCCTAGTAATGCCTATGC |
| rs115645935 | ACGTTGGATGCAGTGGTTTTATACTGAAGG | ACGTTGGATGGGAAGGGAGGAGAAATTAGG | gTACTGAAGGAAAAACACAAG |
| rs75823770 | ACGTTGGATGCAACACAGAGGAGAGAAAAC | ACGTTGGATGTGGTAATAAACGCCTTTAT | AGAGGAGAGAAAACAAAAATA |
| rs75604548 | ACGTTGGATGGTTGACATGAATTAACCTGGC | ACGTTGGATGCTGTTCCTCAAGTCCCATAG | cattAATTAACCTGGCCAAAAA |
| rs77772971 | ACGTTGGATGGTGCACCAAAGGACTTTGGA | ACGTTGGATGGAAAGCCACACTCTGAATAC | TTTGGAATAAAATTCCTGAACC |
| rs115149733 | ACGTTGGATGATCTGGTAGAGAGTCAGGTC | ACGTTGGATGCCACCTTAGTTAGGAGCTAC | cgggCTAAAGGACAGGAAGAGG |
| rs79097024 | ACGTTGGATGTAAGCTGTAATGTTTTGCC | ACGTTGGATGGGTGGACAGACAAACCAGAT | cgcgGTAATGTTTTGCCATGTTG |
| rs1128665 | ACGTTGGATGGTCCTATTGTAAAGAAACGG | ACGTTGGATGGGAAGAGTCCCCATGTAGTA | aggGTAAAGAAACGGAAAAAGTC |
| rs7001041 | ACGTTGGATGGACAAACCCTTGGGGCTTTT | ACGTTGGATGAAGGGCTGCAGCTTGCTCA | cctcGCTTTTTTATTTGGAGAACC |
| rs115544837 | ACGTTGGATGTCCAGGTGTACTAGTGAATG | ACGTTGGATGGCTAGAAGAGAAAATGTACAC | CTAGTGAATGTAATTTATAGTTGC |
| rs77484576 | ACGTTGGATGGTAGATAGGAGCTTATGGTC | ACGTTGGATGCACAACTAGGAAAGACTACAG | agAGGAGCTTATGGTCAAAAAGTG |
| rs75401815 | ACGTTGGATGTCTCTTCCACAGGAAGTTTG | ACGTTGGATGCAAAAGTTCAGGGAAACGGG | cccCCACAGGAAGTTTGGAGCCTAC |
| rs114401825 | ACGTTGGATGCAGTACGAATGTATCTCCTTG | ACGTTGGATGAAATGCCTTGAACACTCTGG | ggCGAATGTATCTCCTTGAAAAATG |
| 52843993 | ACGTTGGATGCATGTCCTCTTCACCAGTGC | ACGTTGGATGTTGCAGGTAGCAGTAGTGTG | caacgCTACACACGTATATTCCACAA |
| 117583864 | ACGTTGGATGAAGTCTAGAGAGGACATTGC | ACGTTGGATGTCCGTGGAGACGTCTTCTTC | cgtcgAGAGAGGACATTGCACCTTTG |
| 69050993 | ACGTTGGATGAAGGCTCGCAAAAAGTCATC | ACGTTGGATGAGAGGAAAGATAAGTGAAC | aagaaTAAATGCATTGTAGCTCTGAG |
| rs114937867 | ACGTTGGATGGTGGGGTTTATAAGGGACTG | ACGTTGGATGTATTTGCAACTCACTGGCCC | ggaatGGACTGAATCAAATGAATGTAA |
| 18631404 | ACGTTGGATGAAATCCTTGCAGCAGAGCAG | ACGTTGGATGAGAAATGCAACGTCGAAGGG | ttttcGAATTTCACAAAAATAAAAGCAG |
| 5026637 | ACGTTGGATGGTGTGAATCAAGTAATTATAC | ACGTTGGATGTTGCCTTTATTTGTAAAGCTG | taAAGTAATTATACAAATAAACATCTGA |
| 25893528 | ACGTTGGATGACACAAGCGGCCCTGACAC | ACGTTGGATGTGACAATGCTGAGGGCTGG | gggaaAAGCGGCCCTGACACGTGATGGA |
| 10973203 | ACGTTGGATGATGAAGGTCTTCCAGAAGCC | ACGTTGGATGTCTCCAACATGATTTCATGC | CAGAAGCCACATCCG |
| 9427681 | ACGTTGGATGTGACGTTCGCCATCATACCG | ACGTTGGATGATGCCGGTCCAAGGTGCAG | ACCTGGTGCCAACTCA |
| 65110509 | ACGTTGGATGTCCAAACATCTTCTGTAGCG | ACGTTGGATGCACTTCATCAGAAAGAGGCG | cCTGTAGCGACCTCACA |
| 39553704 | ACGTTGGATGACTCTGGTCATCTTCGTTCG | ACGTTGGATGCCTCCTGTGGACTAACTTTG | agTCTTCGTTCGTTTGGT |
| 9210798 | ACGTTGGATGTGGCCTCATGTTTCCTTTGC | ACGTTGGATGGCAAACATTTTCACACCCAC | GATCAAGTGGCTTTCAAG |
| 41719240 | ACGTTGGATGAGAGGGTGAAGGTGGGATTG | ACGTTGGATGAGTGAGATTCTGCATGGAGG | gtGGGTAGTTGGGTAGGG |
| 24226155 | ACGTTGGATGTCACCACAATAGCCGTTGAG | ACGTTGGATGACCCGAAGTCAAGAGCTAAG | GAATGACAGAAATGAAGGA |
| rs74621258 | ACGTTGGATGCTGGTTACCAAACATAAATGC | ACGTTGGATGTCCATTCACTTGCAAGCTGG | ACATAAATGCTGAACATTCC |
| rs114313009 | ACGTTGGATGGGAGAACATTTTGCTAAAG | ACGTTGGATGCTCTGCCTTTTTTCCTAAGTC | GCTAAAGCATGACTAAACTG |
| 149683947 | ACGTTGGATGAGGGTTGTTGATGAAGACAG | ACGTTGGATGCTCGCTTTATACCTGAGGAG | cttCTAGGTAGATGGGGAGA |
| rs114408391 | ACGTTGGATGGCCTTAAACTCCTCCTTGAC | ACGTTGGATGAAGTGTTCACCTCAGTTTGG | tgTTTACAAAGATCCAAGCAG |
| rs77085264 | ACGTTGGATGTTAGGGTCTTCCCCCAACAG | ACGTTGGATGCATCTGTGAGCACTGGGAAG | CCCCCAACAGAACTATTTCTTG |
| rs6683364 | ACGTTGGATGCTGGAGCAGTTTCAAAAGCC | ACGTTGGATGGGGAAGAGAATACTGCCTCC | gCTCCAATAACATATCAGCAAT |
| 33040892 | ACGTTGGATGTGTGGTGTAATTGGGATCGC | ACGTTGGATGCTAGCAGGATAACAGATGAG | gtgggTGGGATCGCCCAATAAA |
| 20180291 | ACGTTGGATGACAAAGCAGCCTAATACTTG | ACGTTGGATGGCACAGCCTAACTGATAACC | gGCCTAATACTTGTATTTGAAAC |
| rs117139104 | ACGTTGGATGTTGATCCAGGTGTTTGTTTC | ACGTTGGATGTGTGCAACTGTTGTGGCCAT | TCTAACTTCTGTAATACATACAAT |
| 99511674 | ACGTTGGATGAGTTGAGTGCTACATACAGG | ACGTTGGATGAACTCAACCATGGTCCTTCC | caggGCTACATACAGGATTGAAAC |
| 198368028 | ACGTTGGATGACATGACGAAAGATTTCAG | ACGTTGGATGCTTGGAAAGTACGTAGACTG | gggcCGAAAGATTTCAGAACTTCAG |
| rs1051424 | ACGTTGGATGCAGCAATACTATTTTGCTTT | ACGTTGGATGCACAGGAAAAATAAACGTGG | AATACTATTTTGCTTTAAGTTTTTTT |
| rs117617596 | ACGTTGGATGCATAGTGAGGTATAAAAAAG | ACGTTGGATGGCAAGATGAAGAGGAAAAAC | cctcATTCTTTCTTTTTTTGTGTTTTT |
| rs115002326 | ACGTTGGATGCATGCAGGGTAACATGTAGG | ACGTTGGATGGTGCTTTTTGTTTTCCTTTC | TTCTATATTGAAAGAATACTTTTCTGG |
| rs116276176 | ACGTTGGATGTCTCGAAAAATGGAAGGCTG | ACGTTGGATGGGAAATCCTTTAGACCCGGC | GGCTGTCAATTTAAATTAAATTTATATT |
| rs75377774 | ACGTTGGATGTGTCTCACAGACAGATGTGG | ACGTTGGATGTCAAAGCCATCACAAAACAC | gggtTGGTTCAAAGGTTATCTAAATTTT |
| 2334610 | ACGTTGGATGCTCACGTGTTGCAAGAACAG | ACGTTGGATGACTCCCTCGTAATCAACTAC | CAAGAACAGTTTTGAGCC |
| rs117411065 | ACGTTGGATGTTTCTGGTCATCCCAGGTTC | ACGTTGGATGGGGATTTATCTCCCTCTTGC | cctccCTGACTTTTACCAGGGG |
| rs10817559 | ACGTTGGATGCAAAAAAAATTTACAAAAAAAC | ACGTTGGATGTACCTGTAGAAAACTCTGGC | AAATTTACAAAAAAACAAACACA |
| rs74710244 | ACGTTGGATGGTATCCACTGTTCCAATCTG | ACGTTGGATGCTGTACATGTTTGGTTAAT | CCAATCTGATTTTATTGAAAAGGA |
| rs78268613 | ACGTTGGATGCTCTCATGTTGAACAATCTG | ACGTTGGATGGGATCTTTGACCAGCACTGT | ctgATAAACATTCTCTGAAACCATA |
| rs76046312 | ACGTTGGATGAAGTGGATTATCTGCCTCGG | ACGTTGGATGAAACAGATGCTACCCACAGG | gatgGGGAATTGCTGGTACAAAGAC |
| rs79101693 | ACGTTGGATGGAAGTAGAACTTGAGTTCA | ACGTTGGATGCAGCAGACTCAAGTCAAAGG | AGTTCATGTTTTATATGAAATATTTAC |
| rs115795511 | ACGTTGGATGCAATGGGTGTGGGTAATTTTC | ACGTTGGATGCACAGGAAGGGTTTGTGTAA | TTTCTTAATTTATTTGTAGAAGAGAAG |
|  |  |  |  |
| Target | Assay ID |  |  |
| hsa-miR-15 | #000389 |  |  |
| hsa-miR-16 | #000391 |  |  |
| has-miR-195 | #000494 |  |  |
| has-miR-497 | #0001043 |  |  |
| has-miR-103 | #000439 |  |  |
| has-miR-107 | #000443 |  |  |
| SnU6 | #001973 |  |  |
| VPS4a | Hs002003085_m1 |  |  |
| 18s | Hs99999901_s1 |  |  |
