# Supplementary material for: Identification of a New Target of miR-16, Vacuolar Protein Sorting 4a
Source: PLoS One. 2014 Jul 17;9(7):e101509. doi: 10.1371/journal.pone.0101509 (PMC4102469; doi:10.1371/journal.pone.0101509)
Supplement: Table S5 — Samples utilized in the study. (DOCX) [file pone.0101509.s005.docx]

Table S5. Samples utilized in the study.

| Samples | Use | Repository |
| --- | --- | --- |
| Heart (LV) | |  |
| Non-failing Controls harvested from donor hearts | **RNA** | **Stanford Medical School, CA** |
| HF pre-LVAD harvested at the time of LVAD implant | **RNA** | **University of Minnesota, MN** |
| HF | **DNA** | **University of Minnesota, MN**  **Royal Brompton and Harefield Cardiovascular Biomedical Research Unit, UK** |
| Peripheral blood | |  |
| Healthy Controls | **RNA** | **University of Minnesota, MN** |
| HF pre-LVAD harvested prior to LVAD implant | **RNA** | **University of Minnesota, MN** |
| HF 7 days post-LVAD implant | **RNA** | **University of Minnesota, MN** |
