# Supplementary material for: Identification of a New Target of miR-16, Vacuolar Protein Sorting 4a
Source: PLoS One. 2014 Jul 17;9(7):e101509. doi: 10.1371/journal.pone.0101509 (PMC4102469; doi:10.1371/journal.pone.0101509)
Supplement: Text S1 — Expanded Methods. (DOCX) [file pone.0101509.s006.docx]

**Text S1: SUPPLEMENTAL METHODS**

DNA samples, genotyping and analysis

DNA samples were obtained from individuals (n=855) from the Royal Brompton and Harefield NHS Trust Tissue Typing Service referred for cardiac transplantation due to dilated cardiomyopathy (DCM, n= 234) or ischemic cardiomyopathy (ICM, n= 621), and from individuals at the University of Minnesota (n=152) referred for heart transplant or placement of a LVAD (DCM, n=67 and ICM, n=76). All samples were collected from consenting participants and the study was conducted under the Institutional Review Board approval from the University of Minnesota and the Royal Brompton and Harefield Cardiovascular Biomedical Research Unit, UK. The average age of all patients was 62.4 (standard deviation 11.4), and the cohort consisted of 13.6% females and 86.4% males. SNPs and samples with <95% call rates and SNPs with HWE p-value < 0.001 were excluded from analysis, leading to 138 samples from the University of Minnesota and 812 samples from the Royal Brompton and Harefield NHS Trust Tissue Typing Service.

DNA quality was assessed by combined quality control (QC) at the Biomedical Genomics Facility at the University of Minnesota. Quantitative QC was based on non-allelic quantitative real-time PCR using a single Taqman probe, in order to ensure amplification of DNA samples. Qualitative QC involved genotyping using a balanced polymorphism present in most human populations, in order to ensure that cross-contamination of samples has not occurred.

We identified 853 unique SNPs in 914 unique target sites in 763 unique 3'UTRs in dbSNP. From ESP5500 we identified 343 unique SNPs were found in 348 unique target sites in 311 unique 3'UTRs (starting from an initial size of 14,024,295 SNPs, and 1,837,369 SNPs in the ESP5500 database). Only SNPs with a frequency of at least 1% in the population from dbSNP and all the SNPs from ESP5500 were used. SNPs were prioritized to those within miRNA target sites cognate to miRNA species known to play a role in cardiovascular tissue 1000 genomes, dbSNP and/or ESP5500 also provided estimated population frequencies of these SNPs. Primers as listed in the Table S4 were established for SNPs in miRNA binding sites and samples were genotyped on the commercially available high-throughput genotyping Sequenom MassARRAY platform (San Diego, CA, USA), as we have previously described^1^.

SNPs and samples with <95% call rates and SNPs with HWE p-value < 0.001 were excluded from analysis leading to 138 samples from the University of Minnesota and 812 samples from the Royal Brompton and Harefield NHS Trust Tissue Typing Service. Genotype frequencies of each non-private SNP were compared to that in a general population (Caucasian samples from 1000 Genome project, Phase 1 data, http://www.1000genomes.org/) if possible. We examined distribution of risk alleles in our samples and searched for clustering of risk alleles within samples that deviated from the independence of genotyped loci.

Western Analysis

Total protein from HEK 293T cells 48 h post-transfection was extracted in RIPA lysis buffer as described previously^2^. Total proteins were run on 12% SDS gels (BioRad). Antibodies against VPS4a (1:1000, Sigma) were used to assess protein expression and β-actin (1:1000, Sigma) was used as a loading control. Specific bands of VPS4a and β-actin were detected by using anti-rabbit HRP and anti-mouse HRP secondary antibodies, respectively (Santa Cruz Biotechnology). ECL was used as the chemiluminescence substrate (Pierce). Densitometry was performed to assess the ratio of VPS4a to β-actin band intensity using the ImageJ software.

References

1. Cotsapas C, Prokunina-Olsson L, Welch C, Saxena R, Weaver C, Usher N, Guiducci C, Bonakdar S, Turner N, LaCroix B, Hall JL. Expression analysis of loci associated with type 2 diabetes in human tissues. *Diabetologia*. 2010;53:2334-2339.

2. Adhikari N, Basi, D L, Townsend, D, Rusch, M, Mariash, A, Mullegama, S, Watson, A, Larson, J, Tan, S, Lerman, B, Esko, JD, Selleck, SB, Hall, J L., Basi D, Townsend D, Rusch M, Mariash A, Mullegama S, Watson A, Larson J, Tan S, Lerman B, Esko J, Selleck SB, Hall JL. Heparan sulfate ndst1 regulates vascular smooth muscle cell proliferation, vessel size and vascular remodeling. *Journal of Molecular and Cellular Cardiology*. 2010;49:287-293.
